# Photonic Terahertz for 6G Communication

**DOI:** 10.3390/s26051575

**Published:** 2026-03-02

**Authors:** Jianjun Yu, Ye Zhou

**Affiliations:** State Key Laboratory of ASIC and System, Key Laboratory for Information Science of Electromagnetic Waves (MoE), School of Information Science and Technology, Fudan University, Shanghai 200433, China; zhouye24@m.fudan.edu.cn

**Keywords:** 6G, THz wireless communication, UTC-PD, antenna polarization multiplexing

## Abstract

Terahertz (THz) communication has become a key enabling technology for the future sixth generation (6G) due to its rich spectrum resources, supporting emerging applications such as holographic communication and ultra-wideband transmission. This article provides a comprehensive review of recent advances in photonic THz communications, covering device, system, and antenna technologies. First, the electronic bottlenecks in conventional THz systems, including limited bandwidth and severe phase noise generated by frequency doubling, are discussed, emphasizing the advantages of photonic methods in ultra-wideband signal generation and seamless integration with fiber-optic networks. Then, the effects of the carrier transit time, absorber layer thickness, and saturation effects on the bandwidth efficiency performance in single-row carrier photodiodes are reviewed, as well as multi-parameter co-optimization strategies for an enhanced performance. In addition, the latest progress in high spectral efficiency (SE) multi-dimensional multiplexing, lightweight high-gain lens antennas and multi-antenna MIMO transmission mechanisms in multi-antenna THz systems are also summarized. Finally, the prospects and challenges of photonic THz communications in long-distance links and space applications are discussed.

## 1. Introduction

Over the past decade, wireless data traffic has surged significantly, driven by transformative changes in how modern society generates, shares, and consumes information. The exponential growth is primarily attributed to the widespread adoption of wireless technologies, particularly the proliferation of smart devices such as artificial intelligence (AI), the Internet of Things (IoT) and three-dimensional (3D) media [1]. Since 2020, the fifth-generation (5G) wireless communication networks have been rolled out globally. However, as societal demands evolve, numerous emerging applications have appeared that exceed the capabilities of 5G. For instance, future immersive services such as holographic teleportation call for terabit-per-second throughput and microsecond-scale latency—performance levels unattainable even with 5G’s millimeter wave (mmWave) spectrum. 5G alone is unlikely to fulfill all the demands of future communication systems. Research on sixth-generation (6G) wireless networks is already underway, with large-scale deployment anticipated after 2030 [2,3,4,5,6,7,8,9,10]. To achieve substantially higher data rates, 6G is expected to leverage the terahertz (THz, 0.1–10 THz) spectrum. Among the major technological trends shaping future wireless systems [11,12], THz communication has emerged as a core enabler, offering abundant spectral resources that can support applications such as holographic communication and ultra-broadband transmission. After multiple rounds of coordination among six regional telecommunications organizations worldwide, the 2019 World Radiocommunication Conference (WRC-19) finally approved a total of 137 GHz of bandwidth resources in the 275 GHz–296 GHz, 306 GHz–313 GHz, 318 GHz–333 GHz, and 356 GHz–450 GHz frequency bands for unrestricted use in fixed and land mobile services. This marks the first time the International Telecommunication Union (ITU) has explicitly designated spectrum resources above 275 GHz in the THz band for terrestrial active radio services, and it raises the upper limit of available spectrum resources for active services to 450 GHz, providing fundamental resource guarantees for the development and application of the global THz communication industry.

Currently, two main directions are deployed in THz band signal generation: photonics-based and electronic-based, respectively [13,14,15,16]. Generating mmWave using electrical methods is limited by the bandwidth of electronic devices, resulting in low frequencies, high noise, and problems such as complex equipment structures, large size, and high generation costs. Furthermore, ultra-wideband THz communication faces electronic bottlenecks that limit bandwidth, and its integration with fiber-optic wired communication is complex. Photonic-assisted THz technologies have emerged as a compelling alternative to all-electronic methods, offering unparalleled advantages such as broad frequency bandwidth, tunability and intrinsic stability [17]. Uni-traveling-carrier photodiodes (UTC-PDs) play a central role in photonic THz wave generation, acting as the key optoelectronic interface that enables optical-to-electrical conversion via photomixing. Their performance directly determines the achievable operating frequency, dynamic range, and energy efficiency of the system, thereby making advances in PD technology essential for the progress of THz communication systems [18,19]. Nevertheless, current high-speed photodiodes exhibit a constrained photoelectric conversion efficiency in the sub-THz-frequency regime, which necessitates an elevated optical pump power and additional electrical amplification prior to radiation [20,21]. Such requirements increase overall energy consumption and may introduce nonlinear impairments under practical operating conditions. Consequently, next-generation PDs must simultaneously deliver a high saturation output power, strong responsivity, and broad bandwidth in order to improve both the efficiency and performance of THz communication systems. In Ref. [22], we report a waveguide-integrated modified uni-traveling-carrier photodiode (MUTC-PD) exhibiting an exceptionally high-bandwidth-efficiency product (BEP) exceeding 130 GHz, representing nearly a two-fold improvement over the previously reported state of the art [23]. This performance results from a combination of coordinated design optimizations, including precise engineering of the internal electric-field profile, careful trade-offs in carrier transport dynamics, and the introduction of benzocyclobutene (BCB) beneath the electrode structure to suppress parasitic capacitance. In parallel, a spot-size converter (SSC) is incorporated to improve optical coupling efficiency, thereby enhancing the external responsivity and overall device performance. As a result, the proposed design delivers a record 3 dB bandwidth of 278 GHz for 2 × 7 μm^2^ devices, placing it among the highest values reported for III–V waveguide photodiodes. For larger 2 × 15 μm^2^ devices, a BEP of 133.5 GHz (corresponding to 206 GHz × 64.8%) is achieved, together with bandwidths beyond 200 GHz and an external responsivity of 0.81 A W^−1^. To validate system-level applicability, a WR-5.1 waveguide-packaged MUTC-PD module is developed, delivering output powers above −5 dBm over the 127–185 GHz range and remaining above −10 dBm throughout the entire G-band. Moreover, by exploiting photonics-assisted transmission and dispensing with a low-noise amplifier, a 54 m wireless link supporting data rates up to 120 Gbps is successfully demonstrated, underscoring the strong potential of the proposed devices for future ultra-high-capacity wireless communication systems.

Photonics-assisted approaches offer clear advantages in terms of achievable carrier frequency, frequency tunability, and ultra-wide transmission bandwidth, enabling data rates well beyond 100 Gbps. When combined with advanced digital signal processing (DSP), multiple high-capacity photonics-assisted THz wireless links operating above 300 GHz have been experimentally demonstrated. For example, at a carrier frequency of 339 GHz, a single-channel wireless transmission achieving 124.8 Gbps over a distance of 104 m has been realized through the use of a THz power amplifier, high-gain lens antennas, and probabilistically shaped (PS) 256QAM modulation together with sophisticated equalization algorithms [24]. At 408 GHz, a wireless transmission of 157.46 Gbps over a 10.7 m link has been demonstrated using 16QAM-OFDM modulation combined with offline DSP, including linear equalization, phase-noise mitigation, and nonlinear equalization (NLE) [25]. Nevertheless, to date, there have been no reports of an aggregate information rate approaching 1 Tbps for THz wireless transmission operating in the 330–500 GHz band. Moreover, conventional DSP-based equalization techniques are not well suited for high-order probabilistically shaped QAM signals, which has become a major bottleneck in scaling fiber–THz integrated systems toward terabit-per-second transmission capacities. In Ref. [26], we propose a computationally efficient vector-quantized variational autoencoder (VQ-VAE) architecture integrated with a conventional complex-valued 2 × 2 multi-input multi-output (MIMO) equalizer. This hybrid framework enables simultaneous polarization demultiplexing and nonlinear compensation, while effectively mitigating numerical instability and convergence limitations commonly encountered with high-order probabilistically shaped QAM signals. Leveraging this approach, we demonstrate a dual-channel 2 × 2 MIMO fiber–THz transmission system achieving a record aggregate information rate of 1.0488 Tbps. The system employs 46 GBaud PS-64QAM modulation across the 330–500 GHz band and supports transmission over 10 km of optical fiber followed by a 10 m wireless link, notably without the use of a THz power amplifier.

In THz wireless communication systems, transceiver lens antennas are commonly implemented using plano-convex dielectric structures. As the aperture diameter increases, the physical thickness of such lenses grows significantly, while reducing the lens thickness typically results in an extended focal length. To address these constraints, a Fresnel lens optimized for THz frequencies was theoretically analyzed and numerically designed to simultaneously support a large aperture and a compact focal distance. The electromagnetic characteristics of the proposed THz Fresnel lens, including its performance benefits and inherent trade-offs, were systematically investigated through simulations. To experimentally validate the antenna gain enhancement, a 300 GHz THz wireless communication link was established using the designed Fresnel lens antenna. The measurement results confirm that Fresnel lenses are well suited for long-range THz transmission with large-aperture configurations, effectively alleviating the structural limitations associated with conventional plano-convex lenses. For a Fresnel lens with a diameter of 30 cm, a theoretical antenna gain of 48.83 dB is predicted, while the experimentally measured gain reaches approximately 45 dB at operating frequency of 300 GHz [27].

However, THz signals are highly susceptible to propagation loss and rain attenuation, which significantly limits the achievable transmission distance. In MIMO systems, signal energy can be concentrated into highly directive narrow beams and accurately steered toward the receiver, thereby effectively extending the communication range. Moreover, since antenna dimensions scale inversely with wavelength, compact MIMO antenna arrays can be readily implemented in millimeter-wave systems without imposing excessive space constraints. As a result, MIMO-based mmWave communication is considered a promising solution for future high-capacity wireless networks. Recently, we experimentally demonstrated a W-band radio-over-fiber (RoF) 2 × 2 MIMO mmWave communication system, in which a 93.7 GHz signal is generated using a photonic-assisted approach and recovered through MIMO maximum ratio combining (MRC) and advanced digital signal processing, including the MIMO constant modulus algorithm (CMA) [28]. In addition, the performance of polarization-multiplexed MIMO transmission was experimentally investigated. The results show that the proposed 2 × 2 MIMO system achieves a maximum SNR gain of up to 7.1 dB compared with the 1 × 1 configuration. This work represents the first experimental demonstration of such a high-gain W-band 2 × 2 MIMO millimeter-wave system enabled by photonic-assisted generation and advanced MIMO DSP techniques.

Compared with existing review articles that typically focus on a single layer of the technology stack—such as device physics, system demonstrations, or signal processing techniques—this work provides a vertically integrated perspective spanning the complete photonics-assisted terahertz communication chain. Rather than concentrating solely on photodiode performance metrics, link experiments, or advanced DSP algorithms in isolation, we systematically connect waveguide-integrated MUTC-PD design, multi-dimensional multiplexed system architectures, high-gain lens-enabled link budget enhancement, and intelligent equalization algorithms into a unified framework. This device–system–antenna–algorithm co-design viewpoint enables a comprehensive analysis of how fundamental optoelectronic advances propagate to radiated performance, spectral efficiency, and long-distance transmission capability. By bridging hardware innovation with system validation and signal processing optimization, the review establishes a complete technological pathway toward photonics-enabled 6G wireless infrastructure, distinguishing it from prior surveys that address only partial aspects of the field.

## 2. High-Bandwidth–High-Efficiency UTC-PD Structural Co-Design Method

We report a waveguide-integrated MUTC-PD fabricated on an indium phosphide (InP) platform that effectively overcomes the conventional trade-off between bandwidth and quantum efficiency. The device achieves a 3 dB bandwidth exceeding 200 GHz and an external responsivity of 0.81 A/W, corresponding to a record bandwidth–efficiency product (BEP) of 133.5 GHz. This performance is enabled by synergistic design strategies in optical absorption engineering, carrier transport optimization, and RF impedance matching. From a broader perspective, such a high-BEP photonic THz emitter provides the fundamental hardware capability required for ultra-wideband, high-linearity terahertz signal generation, directly supporting Tbit/s-class wireless links envisioned in photonics-enabled 6G systems and strengthening the bridge between optical fiber infrastructure and next-generation wireless front-ends.

### 2.1. Device Structure and Optical Coupling Optimization

**Figure 1:**
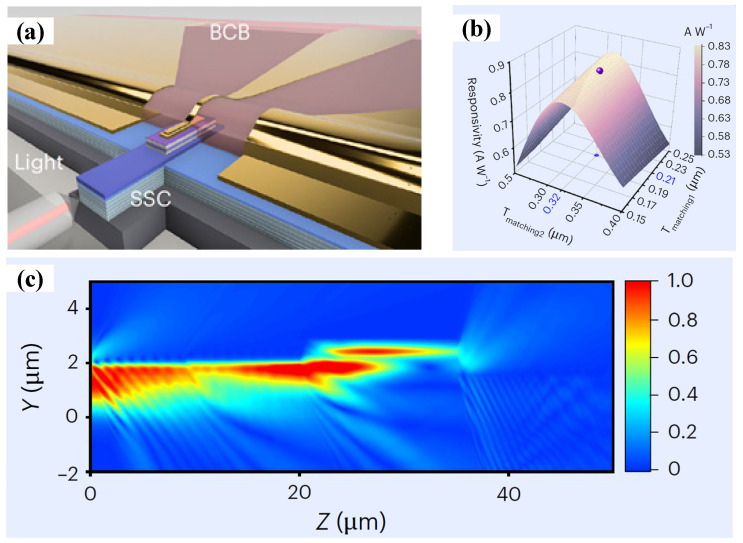
(**a**) Schematic of the device. The integrated SSC efficiently couples the incident optical signal with the active area of the MUTC-PD. The RF output signal is transmitted through a CPW taper to the contact pads of the PD. (**b**) Simulated external responsivity versus thickness of the first (Q1.03) and second (Q1.33) matching layers for the 2 × 15 μm^2^ device. (**c**) Simulated optical intensity as light propagates through the structure. Reprinted with permission from Ref. [22].

Figure 1a illustrates the architecture and operating concept of the proposed waveguide-integrated photodiode chip, comprising a spot-size converter (SSC), an MUTC-PD, and a coplanar waveguide (CPW). To realize efficient optical coupling within a short interaction length, a two-stage SSC is employed, enabling the simultaneous achievement of high responsivity and a rapid RC response. The first SSC segment consists of a 1.5 μm thick diluted waveguide that facilitates efficient fiber-to-waveguide coupling through mode-field matching [29,30,31]. For a Gaussian input beam with a 2 μm spot diameter, numerical simulations indicate a coupling loss as low as 0.2 dB. The second SSC segment incorporates two InGaAsP refractive-index-matching layers that progressively increase the effective refractive index from the diluted waveguide toward the photodiode’s absorption region. This graded transition enhances upward optical confinement and efficient power delivery into an active area as small as 30 μm^2^. The external responsivity of the 2 × 15 μm^2^ device was numerically evaluated as a function of the matching-layer thickness, with an SiO_2_ coating applied to the waveguide facet to suppress Fresnel reflections (Figure 1b). An external responsivity of 0.823 A W^−1^ is achieved when the thicknesses of the first (Q1.03) and second (Q1.33) matching layers are optimized to 210 nm and 320 nm, respectively. The simulated evolution of the optical field intensity along the propagation direction is depicted in Figure 1c.

### 2.2. Epitaxial Structure and Carrier Transport Dynamics Regulation

**Figure 2:**
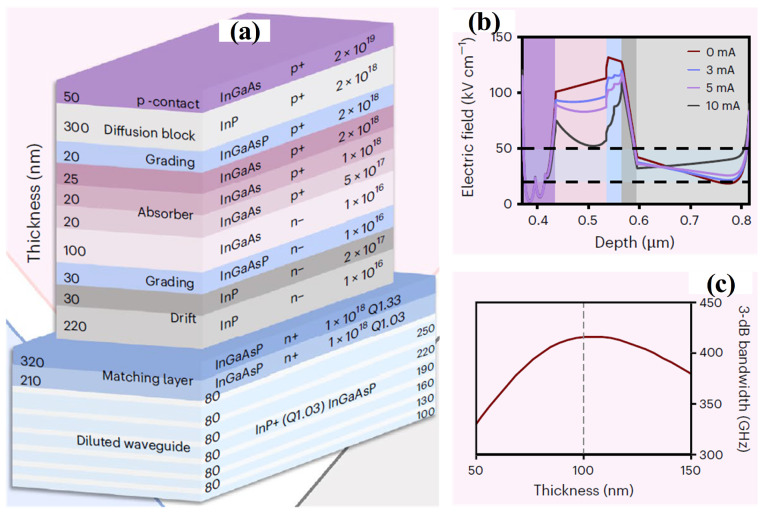
(**a**) Epitaxial layer configuration of the spot-size converter (SSC) and MUTC-PD. The epitaxial stack starts with a diluted waveguide formed by six InGaAsP layers, whose thicknesses gradually increase from 100 nm to 250 nm in 30 nm steps, separated by 80 nm InP spacer layers. Two additional InGaAsP layers positioned above the diluted waveguide function as optical index-matching layers. In the MUTC-PD region, a 165 nm p-type InGaAs absorption layer is partially depleted, leaving a 65 nm undepleted section with a step-graded doping profile (5 × 10^17^, 1 × 10^18^, and 2 × 10^18^ cm^−3^) to establish a quasi-electric field that facilitates electron transport. A 300 nm p-doped InP layer on top serves as the diffusion-blocking layer, while a 250 nm lightly n-doped InP layer beneath acts as the electron drift region. InGaAsP quaternary transition layers are incorporated at the InGaAs–InP heterointerfaces to alleviate band discontinuities. (**b**) Simulated electric-field distributions of the 2 × 15 μm^2^ device under a bias voltage of −1.5 V for different photocurrent levels. (**c**) Calculated transit-time-limited bandwidth as a function of the depleted absorber thickness *W_d_*. Reprinted with permission from Ref. [22].

The cross-sectional schematic of the proposed MUTC structure is presented in Figure 2a. The device incorporates a p-doped, partially depleted InGaAs absorption layer with a thickness of 165 nm, sandwiched between an InP diffusion-blocking layer and an InP drift region. To tailor the internal electric-field profile and promote fast electron transport, a 30 nm thick cliff layer with a doping concentration of 2 × 10^17^ cm^−3^ is embedded within the drift layer. Figure 2b depicts the simulated electric-field distribution of the 2 × 15 μm^2^ device under different photocurrent conditions. The introduction of the highly doped cliff layer significantly enhances the electric field at the InGaAs–InP heterointerfaces (highlighted in blue), thereby lowering the effective interfacial transport barrier and mitigating current-blocking effects [32]. At photocurrents up to 10 mA, the electron drift region (gray area) sustains an electric field in the range of 20–50 kV cm^−1^, which supports electron velocity overshoot [33] and ensures a reduced electron transit time. In addition, the regulated electric-field distribution suppresses space-charge accumulation at elevated current levels, contributing to an improved high-power handling capability. To further minimize carrier transit delay, the thickness of the depleted absorber is set to 100 nm, which is notably larger than the ~30 nm absorber layers commonly employed in previously reported ultra-wide-bandwidth MUTC-PDs [34,35,36]. This configuration effectively suppresses diffusion-dominated carrier transport in the p-type absorber, enabling a larger fraction of photogenerated electrons to be efficiently swept through the high-field depletion region. Figure 2c shows the calculated transit-limited bandwidth as a function of the depleted absorber thickness (*W_d_*), varied from 50 to 150 nm. The analysis is performed using an energy-balance model to accurately capture non-stationary electron transport behavior [37]. As *W_d_* increases, the bandwidth initially improves due to the reduction in electron diffusion time. However, when *W_d_* exceeds 100 nm, the growing influence of slower hole transport within the depleted absorber begins to limit the overall response speed. Consequently, an optimized absorber thickness of 100 nm is selected, yielding a transit-limited bandwidth of 416 GHz. Furthermore, a larger *W_d_* helps maintain a moderate junction capacitance in compact device geometries, which is beneficial for preserving the high-frequency performance.

### 2.3. RF Performance and Parasitic Parameter Management

**Figure 3:**
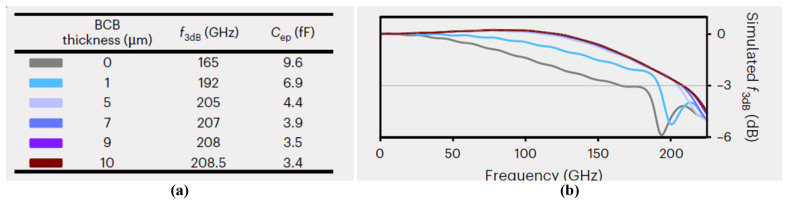
(**a**) Detailed data of the simulated 3 dB bandwidth and *C_ep_* of the 2 × 15 μm^2^ device with varying thicknesses of BCB. (**b**) Simulated frequency response of the 2 × 15 μm^2^ device with varying thicknesses of BCB. Reprinted with permission from Ref. [22].

Beyond carrier transit dynamics and junction capacitance, parasitic effects play a crucial role in determining the achievable bandwidth of the device. To mitigate these limitations, a low-permittivity dielectric material, benzocyclobutene (BCB), is introduced beneath the coplanar waveguide (CPW) electrodes to suppress the electrode parasitic capacitance (*C_ep_*) [38]. This strategy effectively improves the RC-limited frequency response and extends the overall bandwidth. As summarized in Figure 3a,b, the combined influence of RC and transit-time responses, together with *C_ep_*, on the 3 dB bandwidth of the 2 × 15 μm^2^ device was numerically evaluated for different BCB thicknesses. Compared with the conventional configuration in which the CPW electrodes are directly fabricated on the InP substrate, the introduction of a 1 μm thick BCB layer increases the 3 dB bandwidth by approximately 30 GHz. When the BCB thickness is further increased to 5 μm, the simulated 3 dB bandwidth reaches 205 GHz, beyond which only minor additional improvements are observed. Notably, as *C_ep_* is reduced, a weak resonance peak emerges in the frequency response, originating from the intrinsic inductance of the CPW electrodes. This resonance effect further contributes to bandwidth extension. Taking into account both high-frequency performance gains and practical fabrication considerations, the optimal BCB thickness is selected to lie in the range of 5–8 μm.

### 2.4. Actual Performance and Verification of Wireless Communication System

**Figure 4:**
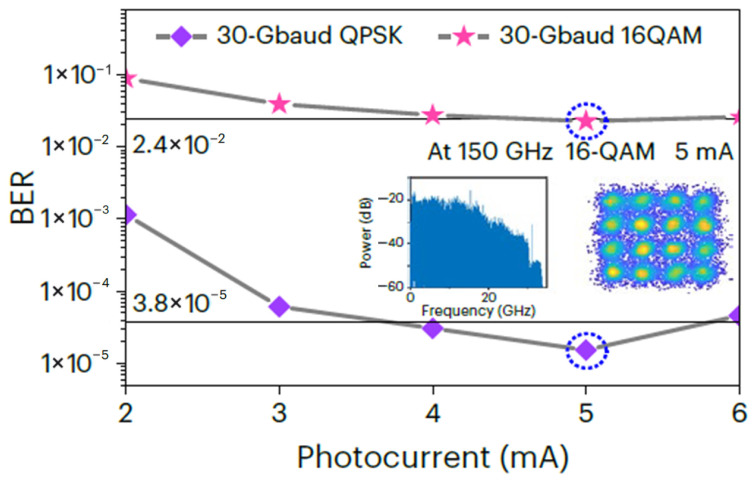
BER versus photocurrent for 30 Gbaud QPSK/16QAM signal at a carrier frequency of 150 GHz. Insets: electrical spectrum (**left**) and constellation diagram (**right**) for a 16QAM signal at 5 mA. Reprinted with permission from Ref. [22].

Figure 4 illustrates the bit error rate (BER) as a function of the photocurrent after 54 m of wireless transmission. As the photocurrent increases, the corresponding RF output power rises, leading to a continuous reduction in BER. At a photocurrent of 5 mA, the measured BERs for 30 GBaud QPSK and 16QAM signals are 3 × 10^−5^ and 2.3 × 10^−2^, respectively, both satisfying the thresholds of hard-decision forward error correction (3.8 × 10^−5^) and soft-decision forward error correction (2.4 × 10^−2^). These results confirm that the proposed system supports a 120 Gbps line rate over a 54 m wireless link without the use of THz amplifiers, demonstrating the enhanced output power and efficiency of the developed MUTC-PD module.

**Figure 5:**
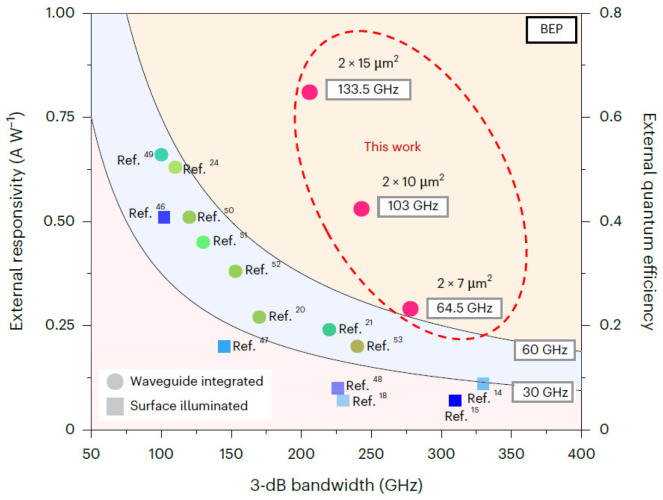
Performance comparison of waveguide-coupled and surface-illuminated PDs. Reprinted with permission from Ref. [22].

Figure 5 summarizes the performance of representative state-of-the-art photodiodes, including both waveguide-coupled and surface-illuminated configurations. While surface-illuminated photodiodes have achieved remarkable operating speeds, with bandwidths extending to approximately 330 GHz, their external responsivity tends to degrade markedly as the bandwidth increases, particularly beyond the 200 GHz regime. In contrast, waveguide-integrated photodiodes separate the optical propagation direction from the carrier transport path, allowing efficient optical absorption to be maintained alongside reduced carrier transit times. As a result, previously reported WG-PDs typically exhibit bandwidth–efficiency products in the range of 37–55 GHz. In the present study, the 2 × 15 μm^2^ device simultaneously delivers a 3 dB bandwidth of 206 GHz and an external responsivity of 0.81 A W^−1^, yielding an exceptionally high BEP of 133.5 GHz (206 GHz × 64.8%). Moreover, the 2 × 7 μm^2^ device achieves a bandwidth of 278 GHz, representing one of the highest values reported to date for waveguide-coupled photodiodes. These performance gains arise from the combined effects of spot-size conversion, optimized carrier transport, and effective suppression of parasitic capacitance.

## 3. Multi-Dimensional Multiplexing High-Spectral-Efficiency Photonic THz Signal Generation Mechanism

Because the gain saturation of a single photoelectric laser-generated carrier constrains the achievable bandwidth under fixed optical power, we propose a multi-dimensional integrated collaborative transmission scheme to enable high-spectral-efficiency photonic THz signal generation. By jointly exploiting multiple dimensions—such as frequency, polarization, and spatial channels—we establish a scalable multiplexing framework that achieves 1.0488 Tbps transmission across the 330–500 GHz band. This approach directly addresses the capacity bottleneck of single-carrier photonic THz emitters and demonstrates a viable pathway toward ultra-high-throughput wireless links. In the broader context of photonics-enabled 6G, this multi-dimensional architecture supports Tbit/s-class data rates and provides a practical strategy for bridging fiber-optic backbone networks with next-generation THz wireless access systems.

It is well established that wireless MIMO techniques can effectively double the transmission capacity in line-of-sight mmWave links, making them a key enabler for future fiber–wireless converged communication systems requiring a high spectral efficiency (SE) and large aggregate throughput. In photonics-assisted mmWave systems, MIMO architectures can be realized through a combination of polarization-multiplexed optical mmWave generation [39], optical multicarrier modulation, antenna polarization multiplexing, high-gain mmWave antennas, multi-band multiplexing, and broadband mmWave signal detection. The integration of these enabling technologies allows the overall data rate to be distributed across multiple spatial, polarization, or frequency channels, thereby reducing the symbol rate and bandwidth requirements imposed on both optical and electrical components. This, in turn, facilitates the practical implementation of broadband photonics-assisted mmWave wireless communication systems.

**Figure 6:**
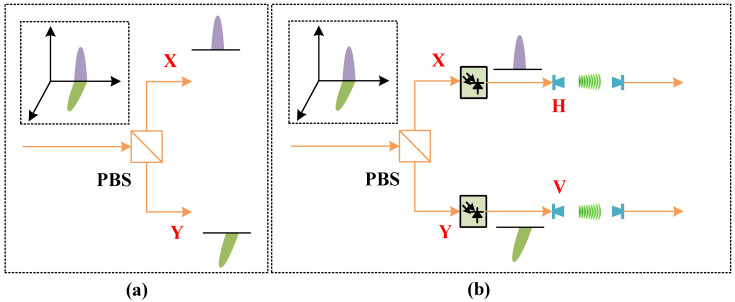
(**a**) Spectra of PDM signals after PBS with optical fiber transmission; (**b**) schematic diagram for polarization-multiplexing mmWave signal based on a MIMO architecture.

As illustrated in Figure 6a, polarization-division multiplexing (PDM) provides an effective means of doubling both the system capacity and the SE. Figure 6b presents the conceptual architecture of a polarization-multiplexed MIMO transmission scheme [40]. The system comprises polarization-multiplexed optical baseband signal generation at the optical transmitter, followed by electrical mmWave signal formation through an optical heterodyne up-conversion process. The resulting signals are then transmitted over a 2 × 2 MIMO wireless link. At the receiver, analog down-conversion is performed prior to offline DSP for polarization demultiplexing and data recovery.

The first step is the generation of polarization-multiplexed optical mmWave signals. This can be achieved by producing two optical mmWave signals with identical optical carrier frequencies but orthogonal states of polarization, which are derived from a common continuous-wave (CW) laser source and subsequently combined using an optical coupler (OC). Alternatively, a polarization-multiplexed I/Q modulator may be employed to directly generate dual-polarization optical mmWave signals [41]. To generate polarization-multiplexed electrical mmWave signals, the incoming dual-polarization optical signals can first be separated using discrete optical components, such as a pair of OCs and polarization beam splitters (PBSs), or alternatively by employing an integrated polarization- and phase-diversity 90° optical hybrid. The separated optical signals are then converted into electrical mmWave signals by two high-speed photodiodes (PDs) [42,43]. The resulting electrical signals form a polarization-multiplexed mmWave signal pair. Wireless transmission of these signals is carried out over a 2 × 2 MIMO link comprising two transmitter antennas and two receiver antennas [44]. At the receiver, the mmWave signals are down-converted to intermediate-frequency (IF) signals using balanced mixers driven by an electrical local oscillator (LO). The IF signals are subsequently captured by a dual-channel real-time oscilloscope, and the recorded waveforms are processed offline using advanced DSP techniques.

Here, the vector (*E_in,x_*, *E_in,y_*)^T^ represents the original optical baseband signal components in the X and Y polarization states generated by the optical baseband transmitter. After optical heterodyne up-conversion, the resulting optical signal can be expressed by the polarization vector (*E_out_*_1*,x*_, *E_out_*_1*,y*_)^T^:

(1) $$\left(\begin{array}{c} E_{out1,x}\\ E_{out1,y} \end{array}\right)=\left(\begin{array}{cc} J_{xx} & J_{xy}\\ J_{yx} & J_{yy} \end{array}\right)\left(\begin{array}{c} E_{in,x}\\ E_{in,y} \end{array}\right)=J\left(\begin{array}{c} E_{in,x}\\ E_{in,y} \end{array}\right)$$

where *J* denotes a 2 × 2 Jones matrix representing the polarization transfer function of the fiber link between the optical baseband transmitter and the optical heterodyne up-converter. The elements *J_xx_* and *J_yy_* characterize the polarization crosstalk between the original X- and Y-polarized signal components introduced during fiber propagation. Subsequently, the mmWa1er can be expressed as

(2) $$\begin{array}{c} \left(\begin{array}{c} E_{out2,x}\\ E_{out2,y} \end{array}\right)=\left(\begin{array}{cc} W_{xx} & W_{xy}\\ W_{yx} & W_{yy} \end{array}\right)\left(\begin{array}{c} E_{out1,x}\\ E_{out1,y} \end{array}\right)\cos wt\\ =W\left(\begin{array}{c} E_{out1,x}\\ E_{out1,y} \end{array}\right)\cos wt \end{array}$$

In this formula, *W* denotes a 2 × 2 gain matrix that represents the transfer function of the 2 × 2 MIMO wireless channel between the optical heterodyne up-converter and the mmWave receiver. The off-diagonal elements *W_xy_* and *W_yx_* describe the polarization and spatial crosstalk introduced during wireless propagation, indicating that each receive antenna may simultaneously capture signals originating from both transmitter antennas. When the transmitter–receiver antenna pairs exhibit high directivity such that each receive antenna predominantly receives the signal from its corresponding transmitter, *W_xy_* and *W_yx_* can be considered negligible. The parameter ω denotes the mmWave carrier angular frequency. By combining Equations (1) and (2), the overall input–output relationship can be expressed as

(3) $$\left(\begin{array}{c} E_{out2,x}\\ E_{out2,y} \end{array}\right)=WJ\left(\begin{array}{c} E_{in,x}\\ E_{in,y} \end{array}\right)\cos(wt)=H\left(\begin{array}{c} E_{in,x}\\ E_{in,y} \end{array}\right)\cos(wt)$$

The overall transfer function *H* of the fiber–wireless link is obtained as the product of the two 2 × 2 matrices and therefore remains a 2 × 2 matrix. As a result, a CMA equalizer employing a 2 × 2 butterfly configuration can be implemented at the receiver to jointly perform polarization demultiplexing and suppression of wireless MIMO crosstalk [45,46].

**Figure 7:**
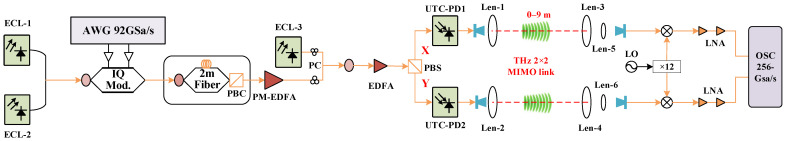
Experimental setup of 1 Tbps THz wireless transmission system.

Figure 7 illustrates the photonic-enabled 1 Tbps fiber–THz 2 × 2 MIMO wireless transmission system. The electrical baseband waveform is generated using a 92 GSa/s arbitrary waveform generator (AWG) featuring an 8-bit vertical resolution and a 32 GHz 3 dB analog bandwidth. Two free-running tunable external-cavity lasers (ECL-1 and ECL-2), operating at optical frequencies of 193.47 THz and 193.565 THz, respectively, are combined and injected into an I/Q modulator as optical carriers. After transmission over 10 km of standard single-mode fiber (SSMF), a third external-cavity laser (ECL-3) at 193.1 THz is introduced as the LO and subsequently amplified by an erbium-doped fiber amplifier (EDFA) to sufficiently drive the UTC-PD. The frequency spacings between Channel 1 (Ch1), Channel 2 (Ch2), and the LO are fixed at 370 GHz and 465 GHz, respectively. Polarization multiplexing is realized by splitting the optical signals into X- and Y-polarization components using a PBS, followed by THz-wave up-conversion via polarization-sensitive UTC-PDs, generating the Ch1 and Ch2 wireless carriers. Two polarization controllers (PCs) are employed to optimize the incident polarization states and maximize the coupled optical power into the UTC-PDs [18]. The two parallel THz signals emitted from the UTC-PDs are transmitted over a 10 m free-space 2 × 2 MIMO link, where three pairs of manually aligned lenses are used to enhance the received THz signal power. At the receiver side, two identical THz receivers operating in the 330–500 GHz band are driven by electronic LO sources to perform analog down-conversion. Each receiver consists of a mixer cascaded with a ×12 frequency multiplier chain, enabling an intermediate-frequency (IF) bandwidth of up to 40 GHz [47]. The LO frequencies for Ch1 and Ch2 are set to 28.83 GHz and 36.75 GHz, respectively. The resulting IF signals are digitized using a 256 GSa/s real-time digital storage oscilloscope with a 59 GHz analog bandwidth and 10-bit resolution for offline signal processing. Although Ch1 and Ch2 are measured independently, the total aggregate information rate (AIUR) of the THz wireless transmitter remains constant at the terabit-per-second level. At the transmitter, a single-carrier 46 GBaud probabilistically shaped (PS) 64QAM optical baseband signal with a roll-off factor of 0.01 is generated by the AWG. For reference, a 46 GBaud 16QAM signal corresponds to a line rate of 736 Gbps. In the implemented system, the measured PS-64QAM modulation achieves an entropy of 5.7 bits per symbol, resulting in a gross line rate of 46 GBaud × 5.7 bit/symbol/polarization × 2 polarizations × 2 channels = 1.0488 Tbps. Assuming an ideal soft-decision forward error correction (SD-FEC) overhead of 25%, corresponding to a threshold BER of 4.2 × 10^−2^ and a code rate of 4/5, the net data rate is calculated as 46 GBaud × [5.7 − 6 × (1 − 4/5)] bit/symbol/polarization × 2 polarizations × 2 channels = 828 Gbps.

**Figure 8:**
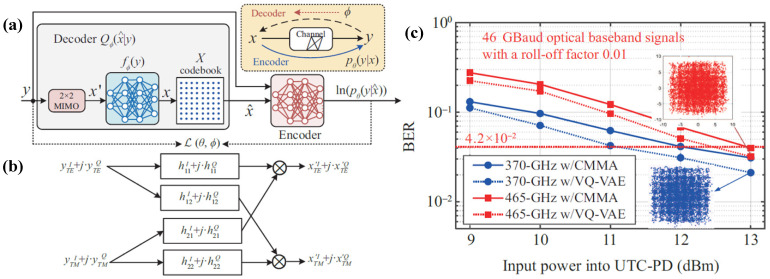
(**a**) Nonlinear compensation using VQ-VAE with (**b**) a classical 2 × 2 butterfly MIMO equalizer. (**c**) BER vs. input power with 46 GBaud PS-64QAM with CMMA and VQ-VAE equalizers at 370 and 465 GHz. Reprinted with permission from Ref. [26].

At the receiver, an efficient vector-quantized variational autoencoder (VQ-VAE) combined with a conventional 2 × 2 butterfly-structured equalizer is employed to perform polarization demultiplexing and channel equalization [48,49], as illustrated in Figure 8a,b. Finally, the BER of the recovered data streams is evaluated to assess the system performance. Based on the optimized parameters, we measure the BER versus input power into each UTC-PD over 10 km SSMF and 10 m wireless distance with 46 GBaud PS-64QAM with CMMA and VQ-VAE equalizers at 370 and 465 GHz, as shown in Figure 8c. A record AIUR of 1.0488 Tbps and a net data rate of 828 Gbps can be achieved. The VQ-VAE method proposed in this study is more robust to variations in frequency space, modulation order, and learning rate, and it can converge to a stable average BER performance under the threshold of 4.2 × 10^−2^. An efficient probability-aware VQ-VAE is used for the recovery of the high-order PS-QAM signals. Unlike traditional adaptive filters (e.g., LMS or CMA), which assume near-Gaussian and uniformly distributed constellations, the VQ-VAE framework can better model nonlinear channel impairments and the non-uniform symbol probability distribution inherent to PS-QAM, thereby improving convergence stability and BER performance under high spectral efficiency conditions. This VQ-VAE equalizer exhibits better convergence properties and nonlinear equalization, which enables the fiber-THz integrated system to achieve Tbps-level THz wireless communication.

## 4. High-Gain Antenna Design Theory

To address the bulkiness of conventional plano-convex dielectric lenses in terahertz transceivers—particularly under large-aperture and short-focal-length requirements—we designed and experimentally validated a terahertz-optimized Fresnel lens. The lens was theoretically analyzed, numerically simulated, and experimentally evaluated in a 300 GHz wireless communication system. The results confirm effective signal enhancement, with a performance approaching that of a standard plano-convex lens: for a 30 cm aperture, the theoretical gain is 48.83 dB, while the measured gain is approximately 45 dB, with deviations mainly arising from alignment tolerances, fabrication precision, installation errors, and material aging. By reducing the weight and structural complexity while maintaining high gain, this Fresnel-lens-based solution supports compact, high-EIRP terahertz front-ends, directly contributing to scalable and cost-effective photonics-enabled 6G systems for long-distance, high-capacity wireless links.

In contemporary THz (THz) transmission systems, dielectric lenses employed at both the transmitter and receiver are predominantly conventional plano-convex lenses [50,51,52,53,54]. These lenses are typically designed based on geometric optics and ray-tracing theory, with the radiation source placed near the focal point of the lens. The lens curvature is determined according to Fermat’s principle and Snell’s law to achieve effective wavefront transformation and focusing. Plano-convex lenses are widely adopted in THz systems owing to their robust focusing performance, broad field of view, and favorable multi-frequency characteristics. In practical deployments, they maintain high antenna gain even in the presence of moderate angular misalignment between the incident wave direction and the lens surface. Furthermore, the design methodology of plano-convex lenses is relatively simple and well established, supported by mature fabrication processes and reliable geometric-optics-based design frameworks. As a result of these advantages, plano-convex lenses have become standard optical components in current THz transmission research, where they are extensively used to enhance antenna gain and directivity, thereby enabling long-distance wireless signal transmission [55,56,57,58,59].

In [54], a research group from Fudan University demonstrated a long-distance THz wireless transmission system with a link distance of 850 m. In the experiment, a 10 cm diameter plano-convex lens was installed at the transmitter to correct phase distortion and collimate the radiated beam, while a 30 cm diameter plano-convex lens was used at the receiver to focus the incoming THz signal. As a result, the effective antenna gains reached 48.5 dB at the transmitter and 65 dB at the receiver. In addition, several studies have highlighted the importance of polytetrafluoroethylene (PTFE) dielectric lenses in long-distance THz links, where antenna gains of up to 70 dB have been reported at both the transmitter and receiver [49,58]. These results clearly demonstrate that high-gain dielectric lenses are essential components for enabling long-distance THz wireless transmission. As the transmission distance increases, THz signals experience greater atmospheric losses and free-space path losses during propagation, necessitating higher gain for lens antennas. Typically, assuming structural losses are negligible, doubling the effective aperture of a plano-convex lens results in an approximate 6 dB increase in antenna gain. Therefore, in long-distance THz transmission, increasing the aperture of the lens antenna becomes a key method for enhancing antenna gain. However, in practice, as the aperture size increases, the volume and weight of the plano-convex lens also increase significantly. Additionally, due to the inverse relationship between the lens focal length and thickness, achieving a balance with short-focal-length large-aperture lenses in engineering practice presents considerable challenges [55,56,57].

To address the aforementioned challenges, Ref. [27] demonstrated a THz lens antenna design—the Fresnel lens. The Fresnel lens consists of a series of concentric prisms etched onto the lens’ base surface, making it lighter and thinner than traditional plano-convex lenses. This characteristic allows the Fresnel lens to maintain a reduced thickness and mass even in scenarios involving large apertures and short focal lengths, thereby helping to minimize material absorption losses and facilitating easier engineering deployment and installation [60,61].

**Figure 9:**
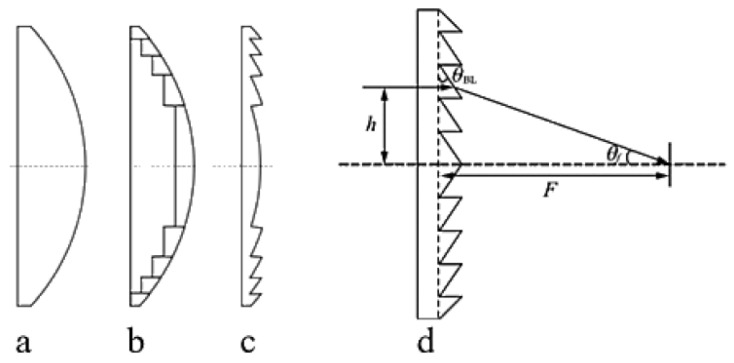
(**a**) Plano-convex lens; (**b**) curved Fresnel lens; (**c**) planar Fresnel lens; (**d**) planar Fresnel lens design schematic. Reprinted with permission from Ref. [27].

The Fresnel lens is derived from the plano-convex (or aspheric) lens. In geometric optics, refraction occurs at the interfaces between different media, and thus the shape of the refractive surface primarily determines the focusing performance. The bulk material between refractive surfaces does not alter the propagation direction of light but increases absorption loss and lens weight [62]. By removing the material that does not contribute to refraction and preserving only the effective refractive zones, the Fresnel lens achieves similar focusing functionality with a significantly reduced thickness and weight, as illustrated in Figure 9.

In practical engineering, due to design and fabrication constraints, the annular surfaces of a Fresnel lens are typically implemented as conical rather than curved profiles. The lens consists of multiple concentric rings, each of which can be regarded, in a cross-section, as a small prism with a specific tilt angle. This approach is therefore commonly referred to as the prism method [63,64,65]. THz waves refracted by these annular prisms are focused at the lens focal point, and the focal length can be tuned by adjusting the tilt angles of the rings. As illustrated in Figure 9d, when considering the refraction of a single annular ring, let *h* denote the incident height, *θ_BL_* the ring tilt angle, *θ_f_* the angle between the refracted ray and the optical axis, and *F* the focal length. Based on Snell’s law, the following relationship can be derived:

(4) $$n\sin\theta_{BL}=\sin(\theta_{BL}+\theta_{f})$$

(5) $$\tan\theta_{f}=\frac{h}{F}$$

The tilt angle of each annular ring and its radial position from the lens center can be determined through iterative optimization. In this work, the lens is designed using the mature optical design software Zemax OpticStudio 19.4, which enables multi-parameter weighted optimization to achieve the desired focusing performance.

**Figure 10:**
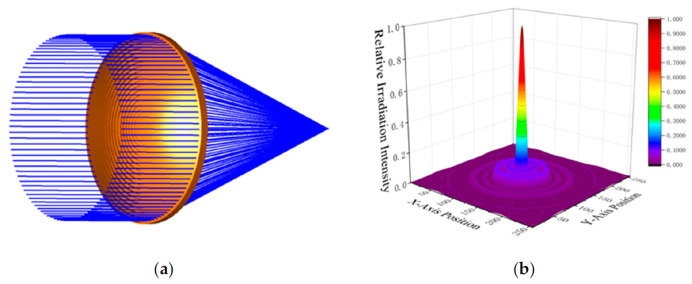
(**a**) Fresnel lens appearance and ray tracing; (**b**) Fresnel lens focal plane radiation intensity. Reprinted with permission from Ref. [27].

A Fresnel lens with an effective aperture of 30 cm was designed for operation at 300 GHz. The lens parameters were iteratively optimized using the prism method and subsequently modeled and analyzed through three-dimensional ray tracing in Zemax OpticStudio 19.4. The simulation results are presented in Figure 10. The relatively high sidelobe levels of the Fresnel lens mainly originate from structural losses. During the optimization process, the number of annular rings was deliberately reduced to accommodate practical manufacturing constraints.

**Figure 11:**
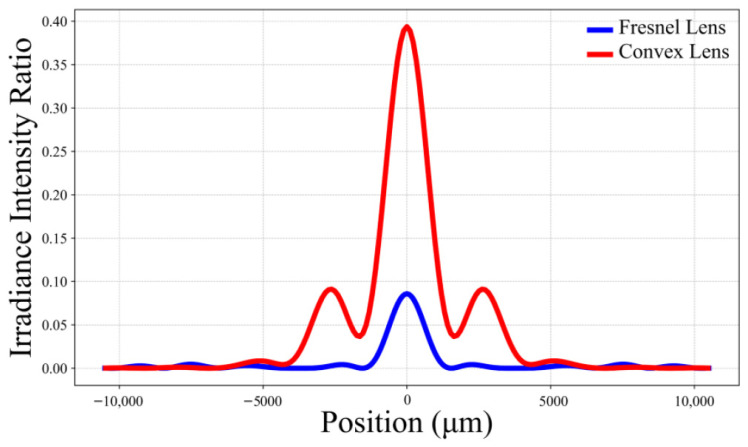
The relative irradiance intensity curves on the focal plane for the two types of lenses. Reprinted with permission from Ref. [27].

Optical simulations were conducted for both a plano-convex lens and a Fresnel lens, each with an effective diameter of 30 cm. The corresponding relative irradiance intensity distributions on the focal plane are shown in Figure 11. The Fresnel lens has a focal length of 35 cm and a maximum thickness of 15 mm, whereas the plano-convex lens has a focal length of 50 cm and a maximum thickness of 55 mm. At a working wavelength of 1 mm (300 GHz), the lens gains were estimated using the Strehl ratio as the peak intensity metric. The calculated gains are 55.44 dB for the plano-convex lens and 48.83 dB for the Fresnel lens, yielding a difference of approximately 6.61 dB. It should be noted that the Strehl ratio primarily reflects imaging quality and can be significantly reduced in the presence of aberrations. As a result, the gain values estimated using this method may underestimate the actual achievable lens gain.

## 5. Multi-Antenna MIMO Transmission Mechanism

Our experiments demonstrate a 7.1 dB gain in a W-band 2 × 2 MIMO system using MIMO-MRC and MIMO-CMA techniques, along with an additional 2.9 dB SNR improvement in a 2 × 2 dual-polarized MIMO configuration. These results confirm that integrating photonic-assisted mmWave MIMO architectures with advanced DSP algorithms significantly enhances link robustness and spectral efficiency, enabling long-range and high-speed wireless transmission. With further incorporation of frequency-division and code-division multiplexing, the system can be scaled toward Tbit/s data rates, and the integration of transmitter-side power amplifiers will support kilometer-level communication distances. Overall, this work aligns with the broader vision of photonics-enabled 6G by demonstrating how photonic front-ends combined with intelligent signal processing can bridge high-capacity optical networks and future mmWave and THz wireless systems.

Due to the limited efficiency of optoelectronic (O–E) converters and the severe penetration loss and atmospheric attenuation in mmWave bands, radio-over-fiber (RoF) mmWave transmission systems based on single-antenna architectures generally suffer from low received power and a degraded signal-to-noise ratio (SNR), particularly at the wireless receiver [66]. To address these limitations and improve wireless link quality, MIMO techniques employing antenna arrays at both the transmitter and receiver have been extensively investigated [67,68]. Most existing MIMO-RoF systems adopt a hybrid MIMO and polarization-division multiplexing (MIMO-PDM) architecture. In such systems, horizontal and vertical polarization channels are typically carried by two spatial MIMO paths, enabling polarization multiplexing with reduced inter-channel interference. Using this approach, we previously demonstrated 10 GBaud PDM-PS-64QAM mmWave transmission at the W-band over a 4.6 km wireless link [69], as well as long-haul 2 × 2 MIMO-PDM transmission achieving 32 Gb/s K-band 16QAM over 2.5 km [70]. Further extensions enabled Tb/s-level wireless transmission in W- and K-bands via photonics-assisted mmWave generation and multi-dimensional multiplexing [71,72], and real-time > 100 Gb/s fiber–THz–fiber transmission beyond 350 GHz [47]. Although MIMO-PDM effectively increases the system capacity, it does not fully exploit the spatial diversity and multiplexing gains inherent to MIMO, as polarization multiplexing primarily relies on orthogonal polarization states rather than spatial channels. To overcome this limitation, we propose and investigate a unipolarized 2 × 2 MIMO system, where multiple transmit antennas radiate the same signal simultaneously. In such a unipolarized MIMO architecture, the key challenge lies in maximizing the received signal gain while maintaining accurate signal recovery. Among various MIMO strategies, maximum-ratio combining (MRC) based on beamforming principles is particularly attractive, as it coherently combines received signals according to their channel gains to achieve diversity enhancement [73]. We previously demonstrated a photon-assisted long-distance SIMO system employing CMA and MRC, achieving up to 80 Gb/s 16QAM transmission at 87.5 GHz with a 1.5 dB improvement in optical power budget [74]. Extending this approach to a 2 × 2 MIMO configuration further enhances the achievable combining and multiplexing gains.

Meanwhile, multipath-induced crosstalk between antenna channels necessitates advanced DSP for accurate signal recovery [75]. Existing MIMO equalization algorithms can be classified into blind and training-based approaches [76]. Blind equalization algorithms, such as the CMA, avoid training overheads but suffer from slower convergence [77], whereas training-based algorithms converge faster at the expense of additional bandwidth consumption [78]. Considering this trade-off, we adopt a blind MIMO-CMA equalization scheme based on a four-butterfly structure.

Using this framework, we experimentally demonstrate a W-band RoF-based 2 × 2 MIMO mmWave communication system. A photonics-assisted technique is employed to generate a 93.7 GHz mmWave signal, which is received using MIMO-MRC and recovered via advanced DSP including the proposed MIMO-CMA-MRC algorithm [28]. For comparison, polarization-multiplexed MIMO systems are also experimentally evaluated. The results show that the SNR gain of the 2 × 2 MIMO system over the 1 × 1 configuration reaches 7.1 dB.

**Figure 12:**
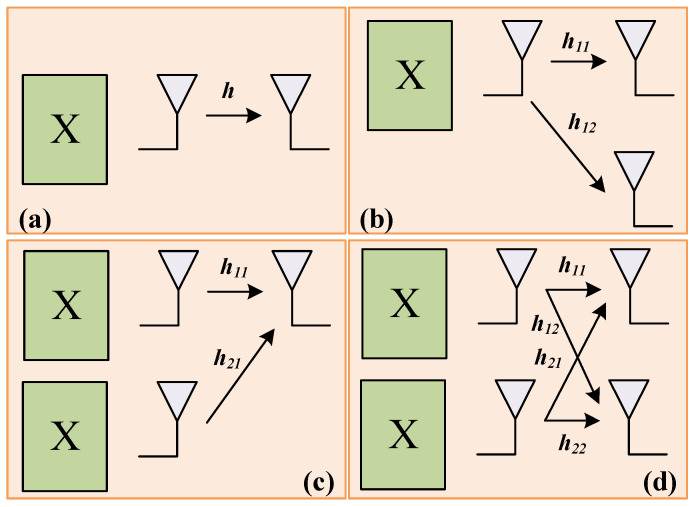
(**a**) SISO system; (**b**) 1 × 2 SIMO system; (**c**) 2 × 1 MISO system; and (**d**) 2 × 2 MIMO system.

As is shown in Figure 12, when the maximum number of antennas is 2, these are SISO, 1 × 2 SIMO, 2 × 1 MISO and 2 × 2 MIMO systems. In the SISO system, the received signal y_SISO_ = hx + n. The SNR of the received signal is

(6) $$SNR_{SISO}=\frac{h^{2}E_{S}}{N_{0}}$$

In summary, the theoretical values of SNR gain for 1 × 2 SIMO, 2 × 1 MISO and 2 × 2 MIMO systems compared to the SISO system are 3, 6 and 9 dB respectively.

**Figure 13:**
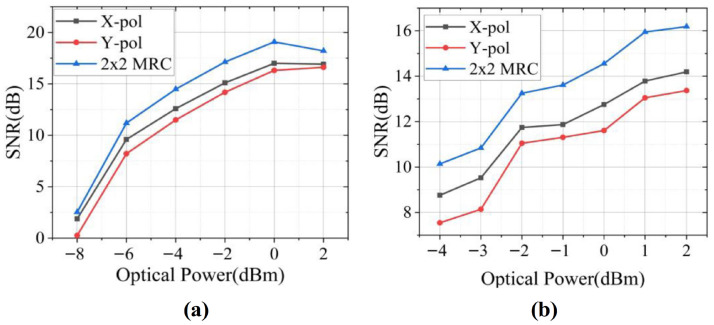
Input power into the PD as a function of SNR for (**a**) 16 GBaud and (**b**) 32 GBaud QPSK signals in a dual-polarization MIMO system after wireless transmission. Reprinted with permission from Ref. [28].

Figure 13a,b present the SNR performance of 16 GBaud and 32 GBaud QPSK signals, respectively, under different transmit powers after 2 m wireless transmission using 1 × 1 V-polarized (X-pol), 1 × 1 H-polarized (Y-pol), and 2 × 2 dual-polarization MIMO configurations. In both baud-rate cases, the 2 × 2 dual-polarized MIMO system achieves an SNR gain of approximately 2.9 dB, which closely matches the theoretical value of 3 dB, with optimal performance observed at an input power of 0 dBm. Compared with H-polarized transmission, V-polarized signals exhibit a superior SNR performance, attributed to the fact that horizontally polarized waves induce polarization currents on the ground surface due to the conductive nature of the earth, resulting in additional attenuation. Moreover, increasing the baud rate from 16 to 32 GBaud leads to a degraded SNR under an identical transmit power, reflecting the increased system bandwidth requirement. When comparing MIMO-PDM with the unipolarized 1 × 1 SISO system using X-polarization, MIMO-PDM shows a better performance at a low SNR, whereas the unipolarized SISO system becomes advantageous at a high SNR. This behavior is mainly caused by polarization–depolarization effects arising from reflection and scattering in practical polarized MIMO channels, which introduce additional power loss.

## 6. Prospects and Challenges

THz communication has emerged as a pivotal enabling technology for 6G wireless systems owing to its abundant spectral resources and its capability to support disruptive applications such as holographic communication and ultra-broadband transmission. This review has systematically summarized recent advances in photonics-assisted THz communication, covering key developments in devices, systems, and antenna technologies.

By revisiting the fundamental limitations of conventional electronic THz systems—including bandwidth constraints and severe phase noise introduced by frequency multiplication—this paper highlights the inherent advantages of photonic approaches in generating ultra-wideband, high-frequency signals and enabling seamless integration with optical fiber networks. In particular, the performance evolution of UTC-PD has been analyzed, with an emphasis on how the carrier transit time, absorption-layer engineering, and saturation effects jointly determine the bandwidth–efficiency product. Recent progress demonstrates that multi-parameter co-design, encompassing optical coupling, energy-band engineering, carrier transport optimization, and parasitic reduction, is essential to overcome the traditional trade-off between bandwidth and responsivity.

At the system level, this review has outlined state-of-the-art THz transmission architectures employing high-order modulation formats, advanced DSP, and multi-dimensional multiplexing. The rapid development of multi-antenna techniques, including polarization multiplexing and MIMO transmission, has significantly improved SE and link robustness. Meanwhile, the adoption of lightweight, high-gain dielectric and Fresnel lens antennas has proven critical for extending the transmission distance while mitigating the size and loss limitations of conventional optics.

Photonics-assisted THz communication is expected to play a pivotal role in 6G, particularly for long-distance wireless backhaul, ultra-high-capacity links, and space-based scenarios; however, several key challenges remain. Atmospheric attenuation caused by molecular absorption and rain significantly limits long-distance THz transmission, requiring optimized transmission windows, high-gain antenna–lens co-design, and adaptive beamforming to enhance link robustness. In addition, improving the optoelectronic conversion efficiency and radiated power, especially for devices such as the UTC-PD, and developing compact, high-efficiency THz amplifiers are essential for extending the transmission distance while maintaining spectral efficiency. A further critical step is the transition from discrete laboratory setups to chip-scale integrated photonic THz transceivers through heterogeneous or monolithic integration of lasers, modulators, photodiodes, antennas, and baseband processing units. Addressing these challenges through coordinated advances in photonics, RF engineering, materials science, and communication theory will be crucial to transforming photonic THz technologies into practical, scalable 6G infrastructure bridging optical and wireless networks.

## Data Availability

Data are contained within the article.
